# WLCD: a dataset of lifestyle in relation with women’s cancer

**DOI:** 10.1186/s13104-023-06458-0

**Published:** 2023-08-22

**Authors:** Alireza Ardalani, Mojtaba Daneshvar

**Affiliations:** 1 Iran University of Science and Technology, Tehran, Iran; 2 Tehran University of medical sciences, Tehran, Iran

**Keywords:** Twitter, Text-mining, Cancer, Women, Lifestyle

## Abstract

**Objectives:**

Social media text mining has been widely used to extract information about the experiences and needs of patients regarding various diseases, especially cancer. Understanding these issues is necessary for further management in primary care. Researchers have identified that lifestyle factors such as diet, exercise, alcohol, and Smoking are associated with cancer risks, particularly women’s cancer. Considering the growing trend in the global burden of women’s cancer, it is essential to monitor up-to-date data sources using text mining.

**Data description:**

We have prepared six independent datasets regarding lifestyle components and women’s cancer: (1) a dataset of nutrition containing 10,161 tweets; (2) a dataset of exercise containing 9412 tweets; (3) a dataset of alcohol containing 2132 tweets; (4) a dataset of Smoking containing 4316 tweets; and (5) a dataset of lifestyle (term) containing 1861 tweets. We also construct an additional dataset: (6) a dataset by summing other components containing 27,882 tweets. These data are provided to discover people’s perspectives, knowledge, and experiences regarding lifestyle and women’s cancer. Hence, it should be valuable for healthcare providers to develop more efficient patient management approaches.

## Objective

Cancer is one of the leading causes of mortality and morbidity worldwide. Growing trends in cancer burden, especially among women, have become a significant global health issue [1]. Lifestyle factors, including unhealthy diet, physical inactivity [2], smoking, and alcohol use [3], are among the risk factors of cancer targeted for primary control. On the other hand, cancer progression and treatments might affect different aspects of lifestyle in cancer patients [4].

Nowadays, the data mining of social media platforms has become an important emerging tool for understanding the experiences and needs of cancer patients. There is a wealth of information available that can be used to gain insight into the patient experience relating to lifestyle patterns [5]. In a previous study, assisted with Twitter data related to breast cancer, researchers identified that physical activity and healthy eating are important factors in symptom management in cases [6]. Another study by analyzing tweets related to site-specific cancers found that physical activity and alcohol consumption are among lifestyle habits that might be associated with liver and breast cancer [7].

By analyzing social media conversations, researchers can identify patterns and trends related to these factors, which can be used to develop targeted public health policies to prevent or manage cancer risk [5, 8]. This approach can also help healthcare providers better address cancer patients’ psychological and emotional needs [9]. By analyzing online discussions, investigators can gain insights into patients’ awareness and identify opportunities for providing proper support and resources associated with lifestyle modification approaches [8, 10].

Social media data mining provides a unique opportunity for public health strategists to understand better people’s attitudes toward the association between lifestyle and women’s cancer and healthcare delivery [11]. By leveraging this information, researchers and healthcare providers can develop targeted interventions that promote healthy lifestyles and improve treatment outcomes, especially among cancer patients [11, 12]. The main objective of this research is to provide Twitter-based datasets containing tweets related to lifestyle and women’s cancer.

## Data description

This study collected tweets related to Women, Lifestyle, and Cancer as a Dataset (WLCD). We have used the following keywords for each section: (1) Lifestyle components including diet (*“diet”, “nutrition”, “eating”, “food”, and “feed”*), physical activity (*“exercise”, “training”, “workout”, “gym”, “fitness”, “yoga”, “aerobic”, “athlete”, “sedentary”,“ jogging”, “running”, “physical activity”)*, alcohol (*“alcohol”, “drink”, “ethanol”, “liquor”, “drunk”*), Smoking (*“smoke”, “smoking”, “cigar”, “cigarette”, “tobacco”, “smoker”, “shisha”, “vape”*), and lifestyle (*“lifestyle”, “lifestyle”*); (2) Women (“*mother”, “women”, “woman”, “female”, “wife”, “wives”, “gynecologic”, “ovarian”, “ovary”, “cervix”, “cervical”, “breast”, “endometrium”, “endometrial”*); (3) Cancer (*“cancer”, “carcinoma”, “tumor”, “chemotherapy”, “radiotherapy”, “chemo”, “cancerous”*). Combinations of keywords were used to reach pertinent search queries and obtain tweets related to lifestyle and women’s cancer. Two independent researchers applied refining search queries and manual review of extracted tweets to ensure the quality and relevance of the queries. Data were gathered from January 1st to December 31st, 2022. Data collection was not limited to location, user, and originality (retweets and quotes included). Tweets were extracted via the Twitter API and presented as multiple datasets, stored at the “Open Science Framework” (OSF: https://osf.io/wc89z/). We have prepared five datasets according to predefined lifestyle components and a cumulative dataset of components. The name of the datasets and the number of tweets are as follows: (1) Diet and women’s cancer (10,161 Tweets, see Table 1, dataset 1); (2) Exercise and women’s cancer (9412 Tweets, see Table 1, dataset 2); (3) Alcohol and women’s cancer (2132 Tweets, see Table 1, dataset 3); (4) Smoking and women’s cancer (4316 Tweets, see Table 1, dataset 4); (5) Lifestyle and women’s cancer (1861 Tweets, see Table 1, dataset 5); (6) The final dataset of lifestyle components and women’s cancer (27,882 Tweets, see Table 1, dataset 6). Datasets contain the tweet’s text, and are prepared in Excel (.xlsx) and text (.txt) formats (see Table 1).

**Table 1:** Overview of data files/data sets

Label	Name of data file/data set	File types (file extension)	Data repository and identifier (DOI or accession number)
*Data set 1*	*Alcohol*	*Text file (.txt)*, *Excel file (.xlsx)*	*OSF (*10.17605/OSF.IO/WC89Z)
*Data set 2*	*Diet*	*Text file (.txt)*, *Excel file (.xlsx)*	*OSF (* 10.17605/OSF.IO/WC89Z
*Data set 3*	*Exercise*	*Text file (.txt)*, *Excel file (.xlsx)*	*OSF (* 10.17605/OSF.IO/WC89Z
*Data set 4*	*Smoking*	*Text file (.txt)*, *Excel file (.xlsx)*	*OSF (* 10.17605/OSF.IO/WC89Z
*Data set 5*	*Lifestyle (term)*	*Text file (.txt)*, *Excel file (.xlsx)*	*OSF (* 10.17605/OSF.IO/WC89Z
*Data set 6*	*Total*	*Text file (.txt)*, *Excel file (.xlsx)*	*OSF (* 10.17605/OSF.IO/WC89Z

## Limitations

- This study assessed only Twitter users that may not represent the general population. Data from other social media, such as Facebook, Instagram, and Reddit, might be needed to have more comprehensive results.
- People may report inaccurate or incomplete information about their lifestyle and health status due to social undesirability.
- Habits and experiences reported by users may be timely, leading to potential misinterpretation.

## Data Availability

The data described in this Data note can be freely and openly accessed on the OSF repository under reference number wc89z https://osf.io/wc89z/. Please see Table 1 for details and links to the data.

## Abbreviations

WLCD: Women Lifestyle and Cancer Dataset
